# Assessment of disposal practices of expired and unused medications among community pharmacies in Anambra State southeast Nigeria: a mixed study design

**DOI:** 10.1186/s40545-019-0174-1

**Published:** 2019-04-16

**Authors:** Iweh Michael, Brian Ogbonna, Nduka Sunday, Maureen Anetoh, Okonta Matthew

**Affiliations:** 1 Department of Clinical Pharmacy and Pharmacy Management, Nnamdi Azikiwe University, Awka, Anambra State Nigeria; 2 Department of Clinical Pharmacy and Pharmacy Management, University of Nigeria, Nsukka, Nigeria

**Keywords:** Expired drugs, Medication disposal, Environment, Community pharmacists, Policy, Nigeria

## Abstract

**Background:**

Expired or unused medicines are potentially toxic substances that should be managed effectively to avoid accumulation of potentially toxic pharmaceuticals in the environment. In Nigeria, there is currently limited literature on the methods and protocols used by community pharmacists in the disposal of expired drugs. This study assessed disposal practices of expired and unused medications by pharmacists in Anambra State and compared them to the National Agency for Food and Drug Administration and Control (NAFDAC) guideline on disposal of expired drugs.

**Methods:**

A questionnaire survey and key informant interview (KII) was used for the study. The pre-tested revised and adapted questionnaires were sent to all the 103 community pharmacies in Pharmacists Council of Nigeria (PCN) 2015 record of registered pharmacies in Anambra State. The participants were asked questions about how they disposed of expired and unused medications. The respondents that used NAFDAC or drug wholesalers were asked questions on how the third party destroys their expired drugs. In addition to the use of a questionnaire, KII was conducted to assess relevant professionals and stakeholders in this area.

**Results:**

The study recorded 77 successfully returned questionnaires out of the 103 distributed and a response rate of 74.8%. The various disposal methods were: through NAFDAC 28.0 (31.8%), drug distributors 21.0 (23.9%), rubbish bins 8.0 (9.1%); this was mainly for solid dosage forms. However, 6.0(7.1%) reported that they used the sink to dispose of their liquid dosage forms and 24.0 (29.6%) noted they do not stock Class B controlled drugs. A lesser proportion of respondents 18.0 (23.4%) complied fully with the national guideline on disposal of expired drugs, while 17.0 (22.1%) complied partially and 42.0 (54.5%) did not comply. Some of the respondents 17.0 (22.1%) reported that NAFDAC uses incineration or other forms of heat to dispose of expired drugs, but 19.0 (24.7%) reported they do not know how NAFDAC dispose of their expired drugs. Majority of the respondents 55.0 (71.4%) suggested the state-run disposal system.

**Conclusion:**

Poor compliance with the national guideline for medication disposal increases the potential risk of contamination of our environment and increases the possibility of ingesting toxic pharmaceutical wastes by humans and animals. This underscores the need for improvement on expired drugs management protocols to prevent contaminations and the attendant health hazards.

## Background

Pharmaceutical products are essential in maintaining human health, but many pharmaceuticals contain hazardous chemicals that can contaminate the environment if they are not properly managed or discarded [1]. When pharmaceutical wastes are improperly disposed of, they can lead to contamination and a wide range of toxicities in man and animals. People can be exposed or accumulate traces or residues of pharmaceuticals from the environment by drinking contaminated water [2]. Many households frequently store unwanted, unused, or expired medications in their homes indefinitely or discard them through general municipal waste bins, sinks, or flush them down their toilets. It is necessary to know that disposing of unused or expired medications through these unauthorized channels predispose the environment and the inhabitants to serious health risks [3]. The wrong disposal of expired pharmaceuticals has led to medication poisoning in adult and children [4]. Narcotic pain relievers and sleep aids found in garbages have the potential of being abused. There is growing concern about the safety of the national water supply, due to recent reports of the detection of antibiotics, antidepressants, and hormone replacement medications in waterways nationwide [4]. Trace amounts of pharmaceuticals and their metabolites have been identified in some drinking water supplies [5].

The presence of expired pharmaceuticals in the sewage can lead to increased antibiotic resistance to the numerous strains of microorganisms found in sewage which can mutate to deadly and resistant pathogens from harmless microbes [6]. Non-biodegradable anti-infective drugs, cytotoxics, and disinfectants cause the destruction of bacteria necessary for sewage treatment [7]. Recently, antibiotics resistance in humans has been traced to the injection of trace quantities of antibiotics in the surrounding waters due to the poor disposal of expired and unused pharmaceuticals [8–10]. Environmental contamination with non-steroidal anti-inflammatory drugs (NSAID) especially diclofenac which has been shown to cause renal failure in vultures following the ingestion of carrion from cattle treated with the drug [11]. Estrogenic compounds used in oral contraceptives like 17-α-ethinylestradiol feminize fishes in minute concentrations leading to infertility [12].

Proper disposal handling and management of drugs prevent avoidable toxicities and promote the safe and friendly environment. The most commonly returned or expired medications are those used in chronic conditions like antihypertensives and anti-diabetes medications usually associated with non-adherence [13]. Unused pharmaceuticals could arise from alterations to the prescribed treatment. Such practices ultimately lead to the expiration of medications which are eventually stored or disposed of by households into the sewerage system, garbages, and refuse dumps or returned and accumulated in community pharmacies [14]. Good medicine disposal practice is an essential aspect of public health preventive services carried out by community pharmacists due to their proximity and accessibility to health seekers in our communities. Community pharmacies are meant to collect, sort, and dispose of expired drugs rationally more than anyone else could. They are meant to serve as collection points for expired drugs in the communities where they are located because they have been trained on the act of proper drugs disposal. It underscores the need for proper medicine disposal in the good interest of our environment and population health. This study assessed the disposal practices of expired and unused medications by community pharmacies and compared them with that of the regulatory agency, the National Agency for Food and Drug Administration and Control (NAFDAC) standard guideline on disposal of expired drugs.

## Methods

### Study area

The study was conducted in Anambra State in southeast Nigeria. It is located in the tropical rainforest belt with an average of 8 months of rainfall annually. It is predominantly a commercial center with numerous small, medium and large scale businesses. The state is surrounded by rivers and susceptible to erosion and flood, which can be contaminated by pharmaceutical waste products. The capital of the state is Awka but has Onitsha and Nnewi as its two major commercial centers. Three senatorial zones (South, Central, and North) in the state encompass 21 Local Government Areas.

### Study population

The population for the study comprised of all registered community pharmacies in Anambra State. There are one hundred and three (103) registered community pharmacies in Anambra State (PCN record, 2015).

### Research design

The study utilized a mixed method research design which comprised of a questionnaire survey and key informant interview.

### Sample/sampling

#### For the questionnaires

In order to account for possible refusals and inappropriate filling of some questionnaires, all the population size (103) registered community pharmacies in 2015 Pharmacists Council of Nigeria (PCN) record were all used. This is also in line with the opinion in a study which showed that when the population size is manageable, there is no need for sampling [15].

#### For the key informant interviews (KIIs)

The 9 Key experts in the field of study were employed in the KIIs. Others include a Senior Staff and two academics in Clinical Pharmacy with more than 10 years working experience). Only registered community and pharmacists who gave their informed consent to participate in the study were included in the survey.

### Preparation, revalidation, and reliability of the instrument

During the first stage of the study, a questionnaire, which was adapted from a study done by Tong et al. in New Zealand, 2011 was used after modifications and revalidation [16]. The instrument was evaluated by seven experts in the field of study who subjected it to face and content validation. The questionnaire comprises of five sections used in obtaining information on respondents’ demography, disposal practices toward unused and expired medications, the role of NAFDAC and drug distributors or drug wholesalers, and questions regarding the funding of state-run destruction system. The questions for the KII were first field-tested on five key informants who did not participate in the final survey and the necessary modifications and corrections were made.

A pilot study was implemented to pre-test the research instrument (questionnaire) for the community pharmacists. This was carried out by giving the questionnaires to 10 registered community pharmacists outside the area who filled them and handed it back the same day. All the community pharmacists who participated in the pilot survey were excluded from the main study. Reliability test analysis (Cronbach’s alpha analysis) was used to test for internal consistency of the instrument. The outcomes of the pilot study were further used to edit the questionnaire to enhance clarity and flow. The key informant interview protocol used in the second phase of the study involved an interview section with questions derived from the outcomes of the first phase of the research questionnaires. It has 7 open-ended questions based on the ways through which the expired drugs were disposed, drug wholesalers/distributors and expired drugs, separation, and storage of expired drugs, school of pharmacies and lectures on disposal practices, challenges to proper disposal practices and the recommendations.

### Ethical approval

Ethical approval to conduct the study was obtained from the research and ethics division of the Ministry of Health Anambra State (Reference number: MH/COMM/523/74).

### Informed consent

Only pharmacists and key informants who gave their informed consent to participate in the study were allowed to participate.

### Data collection

Sequential quantitative and qualitative data collection methods were used. Data collection lasted from November 2016 to April 2017. Quantitative data collection involved the use of self-administered questionnaires that were distributed to 103 community pharmacies across the three senatorial zones in the state. Most of the pharmacists received, filled, and returned their questionnaires. The researchers used the attendance register to figure out the few absentees and then distributed questionnaires to them. The questionnaires were collected during a subsequent visit at their locations after following up through phone calls.

The second stage of the study involved the use of key informant interviews (KIIs) Protocol that comprised of sensitive and relevant questions that were answered by the key experts in the pharmaceuticals and health sectors. Face to face interview and telephone interview technique (which was employed when it was difficult to conduct the face-to-face interview) were employed. During the course of the interview, a tape recorder and note-writing formats were used to collect data.

### Data analysis

All the collected questionnaires were sorted and examined for quality and accuracy before analyzing the data statistically. Demographic variables of the survey (age, gender, length of practice, the senatorial zone of the respondents) were summarized using descriptive statistics of frequencies and percentages of the different disposal practices employed in the community pharmacies. Multiple response-single coding was employed to treat each item as continuous data suited for analysis. The qualitative data obtained from the KIIs were transcribed verbatim, translated where necessary and field notes made.

## Results

### Questionnaire study result

#### Characteristics of the respondents

The demographic characteristics of the respondents indicated that majority (33.8%) of community pharmacists were within the age range of 25 – 34 years and most of them were male (Table 1). The table also indicates the various duration of pharmacy practice among the respondents, which are: 46.8% have practiced less than 10 years, 18.2% have practiced between 11 to 20 years, 20.8% have practiced between 21 to 30 years, and 14.3% have practiced more than 30 years. Table 2 shows the various disposal practices of different community pharmacies in Anambra State. Their various routes of disposal were through; rubbish bin or general municipal waste, sink, toilet, NAFDAC bin, pharmaceutical distributors and burning. However, some (29.6%) did not stock class B controlled drugs (e.g. amphetamine) and 6.0% of the respondents did not stock class C controlled drugs (e.g. benzodiazepines). In general, three most used disposal routes in descending order were; through NAFDAC especially for class C controlled drugs (36.1%), followed by discarding via drug wholesalers/distributors and finally via rubbish bin or general municipal waste. Few respondents burnt their waste on their own and others discarded either through the toilet or through the sink. There was actually no consistency in the respondents’ disposal patterns. Table 3 shows that majority (54.5%) of the participants did not comply with NAFDAC guideline on disposal of expired drugs. Only 23.4% of the respondents complied fully to the guideline. Meanwhile, some (22.1%) respondents complied partially with the guideline. The partial compliance means that those respondents have dual methods of disposal which were via NAFDAC and other disposal means.

**Table 1:** Demographic characteristics of the respondents (*n* = 77)

Variable	Frequency n	Percent (%)
Age of respondents		
24 year and below	4.0	5.2
25–34 years	26.0	33.8
35–44 years	13.0	16.9
45–54 years	17.0	22.1
55–64 years	13.0	16.9
≥ 65 years	4.0	5.2
Gender of respondents		
Male	53.0	68.8
Female	24.0	31.2
Length of practice as a pharmacist		
10 years and less	36.0	46.8
11–20 years	14.0	18.2
21–30 years	16.0	20.8
31–40 years	11.0	14.3
Senatorial zone of respondent		
South	26.0	33.8
Central	26.0	33.8
North	25.0	32.5

## Discussion

This study assessed the disposal practices of expired and unused medications among community pharmacies in Anambra State. The study employed a triangulated method of research, which involved the use of self-administered questionnaires and key informant interview protocols. As such, quantitative and qualitative data collections and analysis were employed in the study. Participation in this research was purely voluntary and the data collected were kept confidential. The ultimate goal of this study was to discover the various ways community pharmacies disposed of their expired or unused medications, determine if their practices were in accordance with NAFDAC guideline on expired pharmaceutical drugs disposal system and the challenges that led to the improper disposal of expired drugs. The need for this research was inspired by poor documentation of the management of expired drugs in Nigeria. According to a study on “Awareness, knowledge, attitudes, and practices of pharmacists toward disposal of unwanted pharmaceuticals”, almost none of the studied medical facilities had documentation or guideline on management of unwanted pharmaceuticals [17].

In the questionnaire survey, the participants were community pharmacists based in Anambra State; other health care workers were excluded. The study made use of an already validated and used the questionnaire in New Zealand [16], which was slightly modified and revalidated to suit the study area. The questionnaire has five sections; section namely: the demographic information of the respondents, the respondents’ disposal practices towards unused and expired prescription and over the counter drugs, and the respondents’ disposal practices for expired and unused controlled drugs. Section 4 was made up of questions related to the role of NAFDAC and drug wholesalers/distributors in medication destruction, while section five was questioned on the funding of state-run disposal and destruction system. The results of the disposal pattern of various dosage forms in Table 2 showed inconsistency in the disposal practices across the various drugs dosage forms. This was in line with another study on knowledge, attitude, and practice (KAP) towards disposal of medicines among healthcare professionals in South India, which revealed that participants were very confused about the proper way of drug disposal, as many countries do not have standard drug disposal protocols [18]. These results supported the deduction made from a research conducted in Ghana on “Assessment of pharmaceutical waste management at selected hospitals and homes in Ghana”, where the survey revealed there was confusion among health workers on the appropriate way to dispose of expired drugs [19].

**Table 2:** Disposal pattern of various dosage forms and controlled drugs by community pharmacies in Anambra State

Variables	SDF, N = 88 n (%)	LDF, *N* = 85 n (%)	SSDF, *N* = 88 n (%)	CBCD, *N* = 81 n (%)	CCCD, *N* = 83 n (%)
Rubbish bin	21.0 (23.9)	19.0 (22.4)	21.0 (23.9)	8.0 (9.9)	14.0 (16.9)
Sink	0.0 (0.0)	6.0 (7.1)	2.0 (2.3)	0.0 (0.0)	0.0 (0.0)
Toilet	0.0 (0.0)	0.0 (0.0)	0.0 (0.0)	1.0 (1.2)	1.0 (1.2)
NAFDAC	31.0 (35.2)	27.0 (31.8)	29.0 (33.0)	24.0 (29.6)	30.0 (36.1)
Drug Wholesalers/distributors	28.0 (31.8)	29.0 (34.1)	29.0 (33.0)	20.0 (24.7)	25.0 (30.1)
Burn	8.0 (9.1)	4.0 (4.7)	7.0 (8.0)	4.0 (4.9)	8.0 (9.6)
Don’t stock	0.0 (0.0)	0.0 (0.0)	0.0 (0.0)	24.0 (29.6)	5.0 (6.0)

Key: *SDF* Solid dosage forms, *LDF* Liquid dosage forms, *SSDF* Semisolid dosage forms, *CBCD* Class B controlled drugs, *CCCD* Class C controlled drugs

The study suggested that the most commonly used disposal route for solid dosage forms, semi-solid dosage forms, class B controlled drugs and class C was via NAFDAC. This result is also in line with that of a similar study conducted in New Zealand by Tong et al., on the disposal practices for expired and unused medications in New Zealand community pharmacies. In the case of New Zealand, contractors of “expired drugs disposal” played a similar role as NAFDAC. The other most commonly used routes are by returning to drug wholesalers/distributors and via general disposal through rubbish bin. The table also reveals that most of the community pharmacies in Anambra did not stock class B controlled drugs and few burnt their expired products on their own. Therefore, the findings in Table 2 suggest that the medication dosage forms also influence the various disposal practices. This is contrary to the outcome of a study in Plateau State, where all the respondents reported that their disposal routes were via rubbish bins alone [20]. As such, the medication dosage forms do not influence their disposal route. But the findings in this study is similar to a study in New Zealand on community and household disposal of expired drugs [21], where their disposal practice was influenced by medications dosage forms, liquid dosage forms were the highest to be disposed of via sinks. Such practice leads to the contamination of waterways with expired pharmaceutical products. Some studies have shown that conventional water treatment protocols failed to remove contaminated pharmaceuticals totally [22, 23].

Evaluation of the disposal practices among community pharmacies concerning NAFDAC guideline in Table 3 shows the general percentage compliance level of community pharmacies concerning NAFDAC guideline, which states that all expired drugs should be reported to NAFDAC. From the table, a majority of the participants did not comply with the guideline on disposal of expired drugs. Some of the respondents complied fully to the guideline. Meanwhile, some respondents complied partially with the guideline. The partial compliance means that those respondents have dual methods of disposal, which were via NAFDAC and other disposal means. This is similar to a study in South Africa, which revealed a major policy implementation gap between the national government and the participants [24]. It corresponds with a study in Anambra State, where observations and interviews conducted in the state revealed a low level of compliance with practice regulations [25]. Some Nigerian researchers that conducted a study among four states (Lagos, Ogun, Niger, and Plateau State) in Nigeria reported low compliance on disposal practices of pharmaceutical wastes. They mentioned that regulatory bodies meant to ensure compliance with pharmaceutical waste management are not well equipped to function effectively as expected [26]. A similar study in Ethiopia revealed low compliance with healthcare waste management among participants [27]. This is homogenous with the findings of a study among seven Nigerian hospitals on healthcare waste management, which portrayed poor handling of healthcare waste [28]. Researchers have suggested an urgent attention from federal and state government to prioritize effective pharmaceutical waste management. Meanwhile, the researchers advocated that regulatory bodies should offer incentives such as free education programs on pharmaceutical waste management in order to boost compliance of pharmaceutical personnel [26].

**Table 3:** Compliance status of respondents to NAFDAC guideline on disposal of expired drugs (n = 77)

Compliance status	n (%)
Noncompliance to guideline	42.0 (54.5)
Partial compliance	17.0 (22.1)
Full compliance	18.0 (23.4)
Respondents’ responses on the need for a State-run medicines disposal scheme	
Compliance status	
Noncompliance to guide line	42.0 (54.5)
Partial compliance	17.0 (22.1)
Full compliance	18.0 (23.4)
Respondents’ responses to the need for a State-run medicines disposal scheme	
Yes	55.0 (71.4)
No	7.0 (9.1)
Don’t know or no comment	15.0 (19.5)
Total	77(100)
Respondents view on the institutions to fund State-run medicines disposal and destruction system (N, 87)	
Institution	n (%)
PCN	21.0 (24.1)
NAFDAC	43.0 (49.4)
Pharmaceutical Companies	17.0 (19.5)
Community Pharmacies	2.0 (2.3)
Anyone	4.0 (4.6)

**Table 4:** Key Informant Interviews (KIIs) Outcomes (*N* = 9)

Question categories	Responses (n)	Description
1. What are the various ways expired drugs are disposed of in Anambra?	Through:	
	(a.) General rubbish bin (4)	Many dispose of via general rubbish bin
	(b.) NAFDAC (3)	Some dispose of via NAFDAC
	(c.) Burning (2)	Few others burn
2. Should drug wholesalers or distributors collect back their expired products?	Yes (3) and No (6)	Majority said “no” and few said “yes”
3. Do community pharmacists ensure proper separation and storage of expired drugs?	Yes (8) and No (1)	Majority said “yes”. But very few said “no”.
4. Will you approve for lectures on pharmaceutical waste management to be incorporated in schools of pharmacy curriculum?	Yes (9) and No (0)	All said “yes”
5. Whether there is any need for improvement in our disposal practices of expired medications?	Yes (9) and No (0)	All said “yes”
6. What are the common challenges to proper disposal practices of expired drugs?	Top on the list are:	
	(a.) Poor education and awareness on management of expired drugs	
	(b.) Poor documentaries of the management of expired drugs	
	(c.) Poor law enforcement	
	Others on the list are:	
	(d.) The stress and protocols involved in returning medications to NAFDAC	
7. What are your recommendations?	Top on the list are:	
	(a.) Creating public awareness on the consequences of improper management of expired medications	
	(b.) Adequate training of health workers on the management of expired drugs	
	Others on the list	
	(c.) Implementation of a local government- run disposal system	
	(d.) There is the need for improved research focus and the government attention on this area	
	(e.) Adequate law enforcement strategies	

Most pharmacists chose NAFDAC as their main route of disposal but did not know how they dispose of the expired drugs returned to their office. However, many said they employed the incineration method and other burning techniques as their pattern of destruction of expired drugs. This report is contrary to the result of the research conducted in New Zealand, where a majority of the participants knew that incinerators destroyed the returned expired drugs. Nevertheless, few participants reported no knowledge of how their contractors dealt with their waste materials. The findings of this study are comparable with some studies in Nigeria that revealed that incineration (or burning) method of disposal is the most prominent method of destruction of healthcare wastes in Nigeria [29, 30]. The people that returned their expired or unused medications to drug wholesalers/distributors knew how they dispose of the returned expired drugs. However, few of the participants believed that they employed incineration technique as their disposal method. This result is similar to that of a study conducted in New Zealand where the majority of the respondents reported no knowledge of how their distributors dealt with their medication waste [16].

The views of community pharmacists on whether to introduce a state-run disposal scheme in response to the question; “Do you think Anambra State needs a state-run medicines disposal scheme accessible to all pharmacies across the state?” was predominantly in the affirmative “yes”. The majority of the participants that answered “yes” preferred the project to be funded by NAFDAC owing to the fact that NAFDAC is the regulatory body entrusted to handle such protocols. However, some reported that such project could also be funded by PCN as a governmental body instituted to govern the affairs of the pharmacists. Few others suggested Pharmaceutical distributors or anyone who has such interest to carter for such project to fund it. The findings of this study show that the majority of community pharmacists desired that a state-run disposal scheme would be introduced to the state. This outcome corresponds with the research conducted in New Zealand, where a majority of the respondents answered “yes” on the need for a state-run medicines disposal scheme [16]. The state scheme is to augment the federal system in the management of expired drugs. Such practice is in conformation with the practice in Sweden where management of expired drug is carried out by a state-run system in connection with their national system but the whole system is supervised and moderated by the Swedish Pharmaceutical Society [31]. This finding is also consistent with another study in the United States of America, where the researchers suggested that more options on standard disposal routes such as the introduction of a community or a state collection system might lead to better disposal practices [32]. Some other researchers have reported that the introduction of a third party intervention in the disposal of unwanted pharmaceuticals will boost efficiency on unwanted medicines management [1]. The outcome of the key informant interview goes further to strengthen the results of the questionnaire survey. The report reveals that the majority of the community pharmacists did not comply with the use of NAFDAC as the choice of disposal route in general. The findings of the key informant interview showed that the major route of expired drug and unused drug disposal was via rubbish bin that was finally thrown into the general municipal garbage as shown in Table 4. This corresponds with studies carried out in Ghana [19, 33], Kuwait [34, 35], India [36], and the United Kingdom [37].

Disposing expired and unused medicines in an improper manner leads to environmental health hazards and pollution of the ecosystem [38]. There was inconsistency in the outcome of the KII when compared to the outcome of the questionnaire about the most used route of expired and unused drugs disposal. The outcome of the questionnaire showed that disposal via NAFDAC collection system is the most predominant route but the KII outcome revealed that expired and unused drugs disposal via rubbish bin was the most predominant. Similar inconsistency has occurred between self-reported attitudes of pharmacists and their actual practice measured in a research study conducted in Turkey [39]. Therefore, it is possible that pharmacists provide more desirable responses in the questionnaires [40]. Majority of the respondents did not support that drug wholesalers or distributors should collect expired drugs from the community pharmacists due to the possibility of re-circulation unless there is a proper regulatory team that sees to the protocols. Majority of the key informants reported that community pharmacists ensured proper sorting and storage of their expired medications pending when it is disposed of. This is in line with the study in Moldova, which revealed that pharmacists were rooted in the basic approach to their practice and they showed a good level of involvement in proper environmental and managerial duties [41].

The position of the key informants on whether to incorporate lectures on “pharmaceutical waste management” in pharmacy school curriculum shows that all the key participants approved on the need to introduce lectures on pharmaceutical waste management in schools of pharmacy curriculum. The general acceptance on the need to introduce this topic in schools of pharmacy curriculum is supported by a study conducted in the United States of America which revealed that people that were taught on standard ways to handle their unused drugs were more able to do so [14]. The relevance of introducing this lectures in pharmacy schools’ curriculum is supported by a study in New Zealand where nearly one-quarter of the respondents stated that the major reason why their expired drugs were kept around the household was that they do not know how to dispose of it [42]. Nigerian researchers have reported that the basic lectures on standard disposal of unwanted drugs are lacking in Nigeria [20]. This can lead to serious consequences on the general safety of the environment due to a lack of basic knowledge on the appropriate method of medication disposal. As such, there is an urgent need to rectify this challenge through education and global scale awareness [43]. Some studies have proven that providing educational interventions on proper disposal practices to pharmacists [44] and student pharmacists [45] produced positive outcomes towards ensuring environmental safety. For the already graduated pharmacists, education on proper disposal practices can be relayed to them via continuing education programs. A study in Egypt has shown that pharmacists are enthusiastic towards continuing education activity [46]. All the key participants reported there is the need for improvement in the area of management of expired drugs in Nigeria. This is not far-fetched from a survey conducted in Lagos where the researchers stated the need for improvement in the area of healthcare waste management in Nigeria [47].

The common challenges on proper disposal practices of expired drugs based on the KII report showed that the major challenges that affect proper disposal of pharmaceutical wastes are; poor education and low level of awareness on the standard protocols in the management of expired pharmaceuticals. Others include limited documentation on this issue, inadequate law enforcement strategies, stress, and protocols involved in returning medications to NAFDAC. This is similar to a study in Gujarat, where the major challenge to proper disposal practices is poor education and low level of awareness on the standard ways of disposal [48]. The researchers, therefore, suggested that improving awareness through educational interventions would boost positive outcomes in this field [48]. A study in California has revealed the positive attitude of pharmacists in educational interventions on drug disposal where necessary, but their knowledge in this area may be poor and they do not disseminate such information consistently to others [49]. Some researchers have mentioned the importance of providing sound education and awareness on the management of expired medicines through various media including newspapers, and television. [50]. Limited documentation on healthcare waste or pharmaceutical waste was also mentioned as one of the challenges. It was supported by some studies in Nigeria [51] and Iran [52]. Indeed, studies have shown that participants dispose of their expired and unused drugs through trash bins [53] mainly because it was easier, less stressful and less time-consuming to them [54]. Therefore, another strategy to boost effective compliance on disposal practices of community pharmacists is by initiating programs or methods that will make standard disposal practice easier and less time-consuming. Lack of adequate legislative policies and enforcement was also one of the challenges mentioned by the respondents, which corresponded with a research study in Afghanistan [55]. As such, there is a need to enhance and strengthen the policies on disposal practices especially for developing countries [56]. Therefore, the recommendations on the improvement are simply the rectifications of these challenges.

## Conclusion

The study showed that community pharmacists in the state employed various routes in the course of disposing of their expired and unused drugs. The outcome of the study revealed an inconsistency in the employed ways of drug disposal. This study identified low compliance to NAFDAC specified standard in the disposal of expired and unused medicines. The result of this study showed that the majority of the community pharmacists that participated in this research desired that state-run disposal scheme that will augment the federal system would be initiated in Anambra State. Perhaps, this will boost efficiency in the management of unwanted pharmaceuticals as observed in various studies previously discussed. The outcome of this study showed that all the various state heads of different pharmaceutical governing bodies that participated in the key informant interview supported the incorporation of pharmaceutical waste management lectures in the school of pharmacy curriculum. They were of the opinion that early educational interventions in this area will ensure better disposal practice, which will promote environmental safety. The major challenges on disposal practices of expired and unused medications revealed in the course of this study were poor education and low level of awareness on the standard protocols in the management of expired pharmaceuticals, limited documentation on this issue, inadequate law enforcement strategies, and protocols involved in returning expired drugs to NAFDAC. The study underscored the need for improvement in the disposal and management of expired and unused pharmaceuticals.

## Abbreviations

ACPN: Association of Community Pharmacists of Nigeria
DPS: Director of Pharmaceutical Services
KAP: Knowledge, Attitudes, and Practices
kii: Key Informant Interview
NAFDAC: National Agency for Food, Drug Administration and Control
NAUTH: Nnamdi Azikiwe University Teaching Hospital
PCN: Pharmacists Council of Nigeria
